# Incentives for Access: Examining Increased Admissions for Cancer Patients After the Patient Driven Payment Model

**DOI:** 10.1093/geroni/igaf122.3966

**Published:** 2025-12-31

**Authors:** Harsha Amaravadi, Rachel Prusynski, Paul Fishman, Natalie Leland, Tracy Mroz

**Affiliations:** University of Washington, Seattle, Washington, United States; University of Washington, Seattle, Washington, United States; University of Washington, Seattle, Washington, United States; University of Pittsburgh, Pittsburgh, Pennsylvania, United States; University of Washington School of Medicine, Seattle, Washington, United States

## Abstract

Metastatic cancer prevalence is increasing as the population ages, highlighting the need for adequate cancer care in skilled nursing facilities (SNFs) which primarily serve older adults. However, cancer care in SNFs has typically been suboptimal. In October 2019, Medicare implemented the Patient Driven Payment Model (PDPM) which aligned SNF payment with patient medical complexity, rather than service volume . While PDPM was not cancer-specific, we hypothesize that PDPM’s incentives impacted SNF patient selection, resulting in increased SNF admissions and altered SNF length-of-stay (LOS) for cancer patients. We used adjusted interrupted time series analysis, leveraging 100% Traditional Medicare claims from 2018-February 2020 to examine SNF admissions pre/post PDPM. Analyses account for patient complexity, discharge-relevant hospital factors, seasonality, and other post-acute care policies. We identified 1,096,484,782 hospital stays for metastatic cancer patients, 13% of which resulted in a SNF stay. We observed opposing patterns for the effect of PDPM on SNF admissions and LOS. For admissions, PDPM was associated with an immediate relative decline of 5.6% in October 2019 followed by a consistent monthly increase of 3.2%. The converse occurred for LOS; we observed an immediate increase (3.8 days, 18%), followed by a consistent monthly reduction of .73 days (-3.6%). Though both total effects (i.e., immediate + monthly trend through February 2020) were positive, the contrasting patterns may suggest changing severity among cancer patients entering SNFs. Findings may reflect PDPM’s financial incentive to admit complex patients, underscoring the need for commensurate care delivery to improve patient outcomes in addition to access.

